# Polyelectrolyte Complexes: A Review of their Applicability in Drug Delivery Technology

**DOI:** 10.4103/0250-474X.58165

**Published:** 2009

**Authors:** S. Lankalapalli, V. R. M. Kolapalli

**Affiliations:** Raghu College of Pharmacy, Dakamarri, Bheemunipatnam, Visakhapatnam 531 162, India; 1 University College of Pharmaceutical Sciences, Andhra University, Visakhapatnam 530 003, India

**Keywords:** Polyelectrolyte complex, polymer, drug delivery, polyion, electrostatic interaction

## Abstract

Over the past several years, great advances have been made towards novel drug delivery systems. The phenomena of interpolymer interactions and formation of polyelectrolyte complexes have been the focus of intensive fundamental and applied research. Interpolyelectrolyte complexes combine unique physicochemical properties with high biocompatibility. Studies have been carried out on many different polymer blends and types. Such combinations may possess unique properties that are different from those of individual component. The present review emphasizes on the applicability of polyelectrolyte complexes in drug delivery technology.

Current state of art is witnessing a revolution in new techniques for drug delivery. These techniques are capable of controlling the rate of drug delivery, sustaining the duration of therapeutic activity and/or targeting the drug to specific tissues. These advancements led to the development of several novel drug delivery systems, revolutionizing the medication with several advantages.

Recent decades witnessed the appearance of polymers that respond in some desired way to changes in temperature, pH, electric or magnetic field. The driving force behind these transitions include stimuli like neutralization of charged groups by either a pH shift or the addition of an oppositely charged polymer, changes in the efficiency of hydrogen bonding with an increase in temperature or ionic strength and collapse of hydrogels and interpenetration of polymer network. These types of polymers not only convert the active substances into a non-deleterious form which can be administered, but also have specific effect on the biodistribution, bioavailability or absorption of the active substances and hence increasingly gaining importance in modern pharmaceutical technology. The interaction between two oppositely charged polymers results in the formation of a complex, termed as polyelectrolyte complex[1]. These polyelectrolyte complexes meet the profile of requirements of biocompatible polymer systems and can be adapted to meet the various requirements like carrier substances and components for active substances.

Polyelectrolyte complexes (PECs) are the association complexes formed between oppositely charged particles (e.g. polymer-polymer, polymer-drug and polymer-drug-polymer). These are formed due to electrostatic interaction between oppositely charged polyions. This avoids the use of chemical cross linking agents, thereby reducing the possible toxicity and other undesirable effects of the reagents. The polyelectrolyte complexes formed between a poly acid and poly base are little affected by the pH variation of the dissolution medium. This concept of complexation, between DNA and chitosan[2], has extensively been studied in the development of delivery vehicle for gene therapy and oral vaccination.

The occurrences of charge-charge interactions between ionic polymers and drugs were considered to be a negative event when the ionic polymers are used as excipients in pharmaceutical formulations. In these systems release of drugs may be strongly affected by the occurrence of charge-charge interactions. However, in recent years these negative events of polymer-drug and polymer-polymer interactions have been exploited positively for controlled drug release[3,4].

## Polyelectrolytes:

The polymers that contain a net negative or positive charge at near neutral pH are called polyelectrolytes[5]. They are generally soluble in water. Their solubility is driven by the electrostatic interactions between water and the charged monomer. Examples of such polymers include DNA, protein, certain derivatives of cellulose polymers and carragenan.

## Classification of polyelectrolytes[6]:

The polyelectrolytes are classified into various types. Based on origin they are classified as natural polyelectrolytes, synthetic polyelectrolytes and chemically modified biopolymers. Based on composition they are homopolymers and copolymers. Based on molecular architecture linear, branched and cross linked. Based on electrochemistry they are classified as polyacids/polyanions, polybases/polycations and polyampholytes. Some of the important polyelectrolytes are exemplified in Table 1.

**TABLE 1:** SOME OF THE IMPORTANT POLYELECTROLYTES

Name	Category (based on the charge type)
Natural Polyelectrolytes	
Nucleic acids	Polyanion
Poly (L-lysine)	Polycation
Poly (L-glutamic acid)	Polyanion
Carrageenan	Polyanion
Alginates	Polyanion
Hyaluronic acid	Polyanion
Chemically modified biopolymers	
Pectin	Polyanion
Chitosan (deacetylation of chitin)	Polycation
Cellulose - based	Polyanion or polycation
Starch - based	Polyanion or polycation
Dextran - based	Polyanion or polycation
Synthetic polyelectrolytes	
Poly (vinylbenzyl trialkyl ammonium)	Polycation
Poly (4-vinyl-N-alkyl-pyridimiun)	Polycation
Poly (acryloyl-oxyalkyl-trialkyl ammonium)	Polycation
Poly (acryamidoalkyl-trialkyl ammonium)	Polycation
Poly (diallydimethyl-ammonium)	Polycation
Poly (styrenesulfonic acid)	Polyanion
Poly (vinylsulfonic acid0	Polyanion
Poly (acrylic or methacrylic acid)	Polyanion
Poly (itaconic acid)	Polyanion
Maleic acid/ diallyamine copolymer	Polyampholytic

## Theoretical aspects of PECs:

Many researchers extensively investigated the properties of the polyelectrolytes[6,7] and the formation of PECs[8–10]. There have been theories proposed based on the electrostatic forces and Flory-Huggins mixing free energies of the polyelectrolytes to explain the mechanism of formation of PECs[11–13]. In general the backbones of the two polymers are not compatible and repel to each other, however, the charge fraction of the polymers determines the type of interaction going to occur between the polymers. When the charge fraction is low, the polymer backbone repulsion (Flory interaction parameter) is dominant and the solution separates in to two phases each containing mostly one of the polymers. At high charge fraction, the attractive electrostatic interactions between the polymers dominate and they precipitate to form a complex. In an intermediate range of charge fraction, the equilibrium state can be a meso phase where the two polymers only separate microscopically. Depending on the stoichiometry of the mixture (the relative concentrations, the relative chain lengths and charge densities), one observes mainly two types of complex formations, a macroscopic phase separation between the solvent and the polymers or a partial aggregation of the polymer chains[14].

## Formation of PECs:

**Fig. 1:**
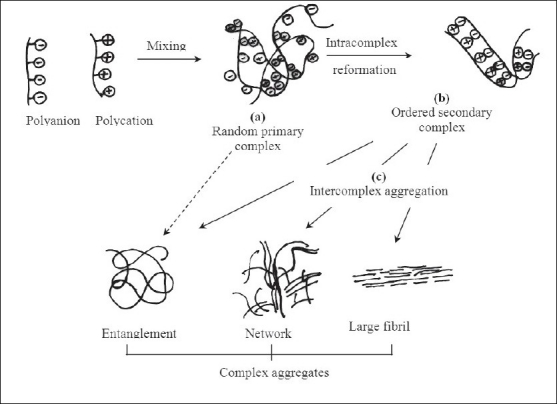
Schematic representation of the formation and aggregation of PECs (a) Primary complex formation (b) Formation process within intracomplexes (c) Inter complex aggregation process

This process involves mainly 3 steps as shown in fig. 1[15]. First step is primary complex formation and Coulomb forces are responsible for this step. Second step is formation process within intracomplexes. It involves formation of new bonds and/or the correction of the distortions of the polymer chains. Third is intercomplex aggregation process, which involves the aggregation of secondary complexes mainly through hydrophobic interactions.

## Factors affecting the formation of PECs:

A number of parameters are known to influence the formation of PECs[16]. These are ion site, charge density, polyelectrolyte concentration, pH, ionic strength, solvents and temperature. Several workers evaluated the factors effecting the formation of polyelectrolyte complexes with different polymeric blends[17,18]. Precipitation is caused by effective attractions due to charge fluctuations and by short range attractions between monomers. Interesting in the context of the polyion stoichiometry are studies of layer formation from strongly asymmetric pairs of polyions. Employing polyions with a reduced charge density along the chain, i.e. consisting of charged and uncharged co-monomers, it was observed that a minimum charge density[19] is required for polyelectrolyte adsorption. Changing the ionic strength by addition of salt[20] can modulate the electrostatic interactions in a polyelectrolyte solution. The electrostatic interactions can be weakened by addition of inorganic salts into the solutions. Thus, an increase of the ionic strength of the solution depresses the complexation between polyions, because of the screening of opposite charges of the macromolecules by low molecular weight ions. By varying the pH environment during PEC formation, the degree of ionization of weak polyelectrolytes can be controlled[21,22]. This was found to affect multilayer properties such as layer thickness, the degree of interpenetration between layers, surface wettability and number of unbound functional groups. Therefore, by choosing the right pH conditions, a platform may be found with properties that are advantageous for loading charged small molecules into the film via electrostatic interactions.

## Characterization of PECs:

Various methods have been used to investigate interactions between polymers[23]. Measurements of turbidity, pH and ionic strength[24,25] as a function of weight ratio of polymer in the media[26], viscosity[27], light scattering[28,29], infrared spectroscopy, NMR, thermal analysis, pK_a_ and powder X-ray diffraction[30] were employed to evaluate interpolymer complexation.

## Applications of PECs:

PECs have gained much attention in the past few years because of their potential applications. These can be used as membranes[31–33], for coating on films and fibers[34], for isolation and fractionation of proteins[35,36], for isolation of nucleic acid[37–39], for binding pharmaceutical products[40], as supports for catalyst[41] and for preparation of microcapsules for drug delivery[42,43]. Many of the applications are based on the functional properties of the polyelectrolyte. The functional applications of PECs are summarized in Table 2.

**TABLE 2:** PRACTICAL APPLICATION OF POLYELECTROLYTE COMPLEXES

Functional property	Application
Interaction with counter ions	Support of filtration process - removal of counter ions[61,62]
	Gelation process - bridging with multivalent counter ions[63]
	Analytical methods - counter ion exchange[64]
Interaction with surfactants	Insoluble polyion surfactant complex for low energy surface modification[65]
	Highly ordered structures (micelle) formation[66,67]
Interaction with charged low molar mass molecules	Polyion drug complexes - soluble or insoluble[68]
Interaction with charged particles	Floculation - waste water treatment[69]
	Dewatering - sludge, pulp and paper production[70]
	Flotation - mining
	Retention - paper production
Interaction with charged surfaces	Displacement chromatography - separation and concentration of biomolecules[38]
	Modification of surfaces and interfaces - coating (antistatic, sensors, multi-layer)[71,72]
	Additive (cosmetics, detergents)

The concept of PECs in the design of drug delivery systems may be useful due to the advancements made during the last two decades[44–47]. The active components will be encapsulated in the polymer matrix at molecular level. They offer greater advantages for the drug substances through improving/and altering physicochemical characters like stability and dissolution. The active substance can be incorporated in to PECs by four ways[48]. In the first case the active substance will be entrapped from the solution during precipitation of the complex. The active substance will be absorbed from the solution and gets incorporated in to the already formed complex on contact in the second case. In the third case the active substance may be chemically bound to at least one complex partner and precipitates during complexation. In the last case the active compound itself may act as poly ion and form PEC. The active substance from these PECs will be released either by solution equilibration or by ion exchange mechanism or by charge interaction and slow decomplexation as well as breakdown and dissolution of the complex.

There are several reports on different methods of preparation and applications of PECs in pharmacy. Kawashima *et al.*[49] developed a novel method for the preparation of theophylline granules coated with a PEC of sodium tripolyphosphate and chitosan. The theophylline granules containing sodium tripolyphosphate were stirred in an HCl solution of chitosan. During the mixing, the dissolved sodium tripolyphosphate in the granules moved to the surface and reacted with the chitosan, resulting in the formation of a PEC film. The drug-release pattern of the coated granules followed zero-order kinetics and the release rates were significantly reduced compared with that of the original granules.

Shiraishi *et al.*[50] studied the controlled drug release behaviour of indomethacin by chitosan-PEC. They also optimized the formulation conditions and reported its *in-vivo/in-vitro* evaluation studies. They prepared the PEC of indomethacin by using complexation of sodium tripolyphosphate and chitosan. Here the effects of the molecular weights of chitosan hydrolysates on the release and absorption rates of indomethacin from gel beads were examined. The release rates of indomethacin decreased with increasing of molecular weight and indomethacin content. A negative correlation was observed between the molecular weight and release rate constant (r=−0.983).

Jimenez-Kairuz *et al.*[51] developed and characterized swellable drug-polyelectrolyte matrices (SDPM) using carbomer and different basic drugs like atenelol, lidocaine and metoclopramide. The authors concluded that drugs can be loaded in a high proportion on to the polymer and therefore the resulting SDPM material could be diluted with other polymers to modulate delivery properties of SDPM. Matrices of atenolol and lidocaine exhibited robust delivery properties with regard to change in proportion of loading drug. Liao *et al.*[52] prepared drug-loaded chitosan-alginate fibers by interfacial polyelectrolyte complexation technique. Depending on the component properties, the release time of encapsulated components from these fibers could range from hours to weeks. Dexamethasone was completely released within 2 h, whereas charged compounds such as bovine serum albumin, PDGF-bb, and avidin showed sustained release for 3 w. In this study, interfacial polyelectrolyte complexation demonstrated to be a promising technique for producing drug-loaded fibers with high encapsulation efficiency, sustained release kinetics, and capacity to retain the bioactivity of the encapsulants.

Tapia *et al.*[53] evaluated the possibility of using mixtures of PECs from both chitosan (CS)-alginate and CS-carrageenan as prolonged release systems. Different dissolution profiles for diltiazem clorhydrate were obtained by changing the polymer matrix system (CS-alginate or CS-carrageenan) and the method used to include these polymers into the formulation (physical mixture or PEC). Drug dissolution profiles from the matrices have been discussed by considering the swelling behavior of the polymers used. They reported that CS-alginate systems were considered to be better in prolonging the release when compared to CS-carrageenan systems.

Paloma *et al.*[54] prepared polyionic complexes of CS and poly(acrylic acid) (PAA) in a wide range of copolymer composition and with two kinds of drugs. Release of amoxicillin trihydrate and amoxicillin sodium from these different complexes was studied. The swelling behavior of and solute transport in swellable hydrogels were investigated to check the effect of polymer/polymer and polymer/drugs interactions. The electrostatic polymer/polymer interactions took place between the cationic groups from CS and the anionic ones from PAA. The diffusion of amoxicillin trihydrate was controlled only by the swelling/eroding ratio of the polyionic complexes. The swelling degree of amoxicillin sodium hydrogels was more extensive when compared to the swelling degree of amoxicillin trihydrate formulations. It was concluded that the water uptake was mainly governed by the degree of ionization. Restriction of amoxicillin sodium diffusion could be achieved by polymer/ionized-drug interaction that retards the drug release. Win *et al.*[55] developed PEC gel beads based on phosphorylated chitosan (PCS) for controlled release of ibuprofen in oral administration. The PCS gel beads were prepared from soluble phosphorylated chitosan by using an ionotropic gelation with counter polyanion, tripolyphosphate (TPP) at pH 4. Surface morphology studies for the prepared beads were done by using SEM. The percentage release of ibuprofen from PCS gel beads was found to be increased as the pH of the dissolution medium increased. The release rate of ibuprofen at pH 7.4 was higher than the release rate at pH 1.4 due to the ionization of phosphate group and higher solubility of ibuprofen at pH 7.4 medium. The ability of the prepared copolymer to be used as drug carrier for colon-specific drug delivery system was estimated using ketoprofen as model drug.

Albeno *et al.*[56] obtained a patent for preparation of stable water insoluble complexes of poorly soluble compounds molecularly dispersed in water insoluble ionic polymers. The compounds were micro precipitated in the ionic polymers in amorphous form. The complexes according to the present invention significantly increased the bioavailability of poorly soluble therapeutically active compounds.

Rolfes *et al.*[57] reported a method of making a solid interpolymer complex for use as a controlled release matrix for oral administration. The process involved mixing of two oppositely charged polymers and spray-dried to evaporate the solvent and to prepare solid particles of interpolymer complex. An active agent such as drug can be preferably embedded or encapsulated in the interpolymer complex before spray drying or may be incorporated by suitable means at a later stage. Mi *et al.*[58] employed enzyme hydrolyzed CS to prepare CS tripolyphosphate and CS polyphosphoric acid gel beads using a polyelectrolyte complexation method for the sustained release of anticancer agent, 6-mercaptopurine. Nandini and Cherng-Ju[59] developed drug PECs with poly(acrylamido-2-methyl-1-propansulfonate sodium-co-methyl-methacrylate. They studied and reported that the release kinetics were strongly dependent on the drug solubility rather than on the type of amine in the drug. The release of drugs from the tablets of drug-poly(acrylamide-2-methyl-1-propane sulfonate sodium-co-methyl methacrylate complex were well described by the dissociation/erosion mechanism. Petzold *et al.*[60] prepared different PECs from poly(diallyl-dimethyl-ammoniumchloride) and two different polyanions and characterized their application as flocculants. The results showed that the most important advantages of PEC were the high velocity of sedimentation and a very broad range of the optimum flocculation concentration.

The review summarized emerging popularity and valuable potential offered by the polyelectrolyte complexes in the field of drug delivery. They represent an attractive class of polymer-based materials finding an irreplaceable role in many areas of the everyday life used for the preparation of biodegradable and biocompatible three-dimensional membranes, microcapsules, nano-sized formulations and various types of controlled release drug delivery systems.

## CONCULSIONS

An extensive research is going on in the area of polyelectrolytes and polyelectrolyte complexes. There is a great potential in utilizing these PECs in ecology, biotechnology, medicine and pharmaceutical technology. However these techniques are not effectively applied for the development of drug delivery systems. They may efficiently modify the release; improve the stability and character of the drug substances due to their capacity to entrap the drug at molecular level. Hence the polyelectrolyte complexes have great potential in the design of novel drug delivery systems.
